# Oxidative stress and inflammation in retained placenta: a pilot study of protein and gene expression of GPX1 and NFκB

**DOI:** 10.1186/s12884-016-1135-1

**Published:** 2016-12-06

**Authors:** Margit Endler, Sissel Saltvedt, Mohamed Eweida, Helena Åkerud

**Affiliations:** 1 Department of Clinical Science and Education, Karolinska Institutet, Södersjukhuset, Stockholm, Sweden; 2 Department of Obstetrics and Gynecology, Karolinska University Hospital, Stockholm, Sweden; 3 Department of Women’s and Children’s Health, Uppsala University, Uppsala, Sweden; 4 Department of Obstetrics and Gynecology, Södersjukhuset, Sjukhusbacken 10, Stockholm, 118 83 Sweden

**Keywords:** Retained placenta, Postpartum hemorrhage, Oxidative stress, Inflammation, GPX, NFκB

## Abstract

**Background:**

Retained placenta is associated with severe postpartum hemorrhage. Its etiology is unknown and its biochemistry has not been studied. We aimed to assess whether levels of the antioxidative enzyme Glutathione Peroxidase 1 (GPX1) and the transcription factor Nuclear Factor κβ (NFκβ), as markers of oxidative stress and inflammation, were affected in retained placentas compared to spontaneously released placentas from otherwise normal full term pregnancies.

**Methods:**

In a pilot study we assessed concentrations of GPX1 by ELISA and gene (mRNA) expression of GPX1, NFκβ and its inhibitor Iκβα, by quantitative real-time-PCR in periumbilical and peripheral samples from retained (*n* = 29) and non-retained (*n* = 31) placental tissue.

**Results:**

Median periumbilical GPX1 concentrations were 13.32 ng/ml in retained placentas and 17.96 ng/ml in non-retained placentas (*p* = 0.22), peripheral concentrations were 13.27 ng/ml and 19.09 ng/ml (*p* = 0.08). Retained placental tissue was more likely to have a low GPX1 protein concentration (OR 3.82, *p* = 0.02 for periumbilical and OR 3.95, *p* = 0.02 for peripheral samples). Median periumbilical GPX1 gene expressions were 1.13 for retained placentas and 0.88 for non-retained placentas (*p* = 0.08), peripheral expression was 1.32 and 1.18 (*p* = 0.46). Gene expressions of NFκβ and Iκβα were not significantly different between retained and non-retained placental tissue.

**Conclusions:**

Women with retained placenta were more likely to have a low level of GPX1 protein concentration in placental tissue compared to women without retained placenta and retained placental tissue showed a tendency of lower median concentrations of GPX1 protein expression. This may indicate decreased antioxidative capacity as a component in this disorder but requires a larger sample to corroborate results.

## Background

Excessive blood loss after delivery is globally the main cause of maternal mortality [1]. Retained placenta may be one of the main causes of death due to hemorrhage in low income countries but its pathophysiology is largely unknown [2, 3].

Retained placenta is diagnosed if the placenta has not detached within 30 min of delivery and occurs after 2–3% of deliveries. Manual removal is usually required to achieve placental separation or in response to excessive bleeding [4, 5]. Studies within veterinary medicine have shown that oxidative stress and inflammation is increased in the placenta of dairy cattle with retained placenta [6]. The pattern of increased oxidative stress and systemic inflammation is also seen in human pregnancies complicated by recurrent miscarriages, preterm delivery, small for gestational age birth and preeclampsia, disorders characterized by an initial defective placentation [7–9]. Epidemiological studies have indicated that retained placenta is associated with these pregnancy disorders [10, 11]. Whether oxidative stress and/or inflammation is part of a common underlying pathological mechanism in both retained placenta and defective placentation disorders remains to be investigated.

Glutathione peroxidase (GPX), a selenium-dependent antioxidant enzyme, plays an essential role in cellular antioxidative defence. GPX1 is the main cellular isoform of the enzyme and is responsible for approximately 80% of enzyme activity in placental tissue [12]. Several studies have shown that GPX1 enzyme activity and/or mRNA expression is decreased in cases of preeclampsia [13–15].

NFκB (nuclear factor kappa-light-chain-enhancer of activated B cells) has been implicated both in the regulation of oxidative stress levels and inflammatory response in the placenta [16, 17]. GPX1 is upregulated by NFκB in response to oxidative stress and there are indications that NFκB gene loci are activated in preeclamptic placenta [18, 19]. NFκB forms a complex with IκBα (nuclear factor of kappa light polypeptide gene enhancer in B-cells inhibitor, alpha) in its inactive state. Phosphorylation of IκBα initiates its dissociation from NFκB which is thereby activated.

To our knowledge, no aspect of the biochemistry of retained placenta in humans has been studied before. This was a pilot study, the aim of which was to assess protein- and gene expression of GPX1 and NFκB, as potential markers of oxidative stress and inflammation, in retained placentas compared to spontaneously released placentas from otherwise normal full term pregnancies. We hypothesized that retained placental tissue would, like preeclamptic placentas, show signs of decreased antioxidative capacity and increased inflammation.

## Methods

A cross-sectional study based on prospectively collected placentas between February 2013 and September 2014 was performed. The study group was recruited from a tertiary level obstetric department with approximately 7500 deliveries per year and a rate of placental retention of 2.6% [20]. The total study group consisted of 29 retained placentas and 31 spontaneously released placentas. All placentas came from live full term singleton births with no clinical indications of preeclampsia, diabetes or fetal growth restriction. Sixty-two women were initially recruited to the study. Two cases of retained placenta were excluded from the study group because of unrecognized diabetes in the mother and clinically suspected placenta accreta respectively.

Retained placenta was defined as the identification of an adherent plane between the decidua and the endometrium during manual removal of the placenta, thus excluding cases where the placenta is trapped behind a closed cervix. Within a few days of each retained placenta being included in the study, a randomly selected delivery with spontaneous release of placenta within 30 min of delivery was selected from the in-patient list for inclusion in the study.

### Sample collection and storage characteristics

Two full thickness peripheral and periumbilical placental biopsies were collected immediately after placental delivery at standardized sampling sites 1–2 cm from the umbilical insertion and 1–2 cm from the periphery respectively. Samples were frozen at −70 °C within 10 min of delivery. The time duration from delivery of the placenta until the samples were frozen was recorded. Variables relating to reproductive history and current delivery, and data relating to the newborn, were recorded for each woman admitted to the study.

The median time from delivery of the placenta till the samples were frozen at −70 °C was 5 min 15 s for retained placentas and 4 min 38 s for non-retained placentas (*p* = 0.4).

### RNA extraction

Total RNA was extracted from each placental sample using Bullet Blender Homogenizer (Next advance Inc., USA) and Aurum total RNA mini kit (Bio-Rad Laboratories Inc., USA). One hundred 100 milligrams of placental tissue was placed in a 1.5 ml sterile screw cap tube (Axygen Scientific, USA) with 100 μL of RNAse free Beads (Next Advance Inc., USA) with 700 μL lysis buffer and β-Mercaptoethanol (Bio-Rad Laboratories Inc., USA). Homogenization time in the Bullet Blender (Next advance Inc., USA) was ten minutes at the highest speed. Tubes were then centrifuged in an Eppendorf centrifuge, 5415 R at 13,000 rpm for 20 min at 4 °C. The total RNA was extracted from the supernatant according to the manufacturer’s instructions. Twice the amount of recommended DNase1 was used to ascertain no remaining cDNA. The quantity of total RNA was measured using the Pico Drop spectrophotometer (PicoDrop LTD, UK). Five micro litres of each RNA sample were analyzed using 1% Agarose gel electrophoresis in TAE buffer and visualized using ethidium bromide staining.

### Real-time quantitative reverse transcriptase polymerase chain reaction analysis (qRT-PCR)

iTaq™ Universal SYBR® Green One-Step Kit (Bio-Rad Laboratories Inc., USA) was used for both cDNA synthesis and real-time PCR analysis of GPX1(1), NFκB and IκBα. The sequences of specific primers used to measure the gene expression of GPX1(1) and housekeeping gene, tyrosine 3-monooxygenase/tryptophan 5-monooxygenase activation protein zeta (YWHAZ), are shown in Table 1. Prime PCR SYBR Green Assay: NF-κB and IκBα (Bio-Rad Laboratories Inc., USA) were used as primers for real-time PCR analysis of NF-κB and IκBα. The specific primer sequences for NF-κB and IκBα were not divulged by the manufacturer (Bio-Rad Laboratories Inc., USA).

**Table 1:** Sequences of primers used to measure gene expression of GPX 1(1) and YWHAZ in retained and non-retained placenta and applicon sizes for GPX1(1), YWHAZ, NF-κB and IκBα mRNA

Gene	Primer	Applicon size
GPX1(1)	F: 5′ TTCCCGTGCAACCAGTTT 3′	63
	R:5′AACGAAGAGATTCTGAATTCCCTC 3′	
YWHAZ	F:5′GCAATTACTGAGAGACAACTTGACA3′	96
	R: 5′ TGGAAGGCCGGTTAATTTT 3′	
NFKB	PrimePCR SYBR Green Assay: NFKBA^a^	76
NFKBIA	PrimePCR SYBR Green Assay: NFKBIA^a^	64

^a^specific primer sequence not divulged by manufacturer (Bio-Rad Laboratories Inc., USA)

Ten μL of iTaq Universal SYBR green reaction mix was mixed with 0.25 μL of iScript reverse transcriptase, 50 pmol of each primer (forward and reverse) and 132 ng of total RNA. The volume was then raised to 20 μL with RNAse free water. The cDNA synthesis and real-time PCR were performed in the PCR system CFX Connect (Bio-Rad Laboratories Inc., USA) according to the manufacturer’s instructions. The real-time PCR products were visualized in 2% agarose gel electrophoresis and ethidium bromide staining.

Each sample was analyzed in duplicates and the mean value of each sample was used for quantification. The formula of Livak and Schmittgen [21] was used for quantification of samples and normalization was performed using the housekeeping gene YWHAZ.

### Enzyme Linked-Immunosorbent Assay (ELISA)

Sandwich enzyme linked-immunosorbent assay (ELISA) was used for quantitative determination of the intracellular GPX1(1) protein in placental samples using the GPX1 human intracellular ELISA kit (Adipogen International Inc., USA). All samples were analysed in duplicates and the researcher was blinded to outcome group. One hundred 100 milligrams of placental tissue was rinsed with 1X PBS. The tissue was placed in an Eppendorf tube with 500 μl of ice-cold homogenization buffer containing 1xPBS pH 7.2, supplemented with a protease inhibitor (complete EDTA-free protease inhibitor cocktail, Roche, Germany), 0.05 Sodium Azide and 0.5% Triton-X-100. The tissue was sonicated on ice for 3 × 10 s (Branson Sonifier 150, USA). The homogenate was incubated for 30 min at 4 °C and centrifuged at 12,000 × *g* for 15 min at 4 °C in an Eppendorf microcentrifuge. The supernatant was assayed for total protein content using DC Protein assay kit (Bio-Rad Laboratories Inc., USA), according to the manufacturer’s instructions. One hundred microliters of the supernatant was loaded to a polyclonal GPX1(1) antibody-specific coated well of the ELISA plate. Each 100 μl from each sample contained an equal amount of total protein. The ELISA procedure was performed according to the manufacturer’s instructions (Adipogen International Inc., USA). A standard curve was performed. GPX1(1) protein standard dilutions were between 4 and 0 nanogram per millilitre. The absorbance was measured at 450 nm using Ascent software v. 2.6. (Thermo Labsystems, Finland).

## Statistical analysis

Medians and interquartile range for non-normally distributed data were calculated groupwise for each marker studied. Comparisons between groups were performed using the Mann-Whitney *U*-test. Categorization into high and low GPX concentration categories was determined based on optimal sensitivity to specificity cut-off in an ROC curve. Odds ratios were calculated using unconditional regression analysis. Correlations were analyzed using Spearman’s rank correlation coefficient. The null hypothesis was rejected where *p* < 0.05. Study size was planned to achieve an 80% probability of detecting a 30% difference in expression of GPX-1 protein between retained placenta and non-retained placentas at 0.05 significance level. Statistical analysis was carried out using the PASW Statistics 18.0.0 (SPSS Inc., 233 South Wacker Drive, 11th Floor Chicago, IL, 60606-6412). The dataset upon which the results if the study rely can be viewed in Attachment 1.

## Results

### Clinical characteristics

There were no significant differences between women with and without retained placenta in regards to age, previous parity, history of previous miscarriages, abortions or caesarean sections, use of assisted reproductive therapy, gestational age, epidural use, induction of labor and instrumental delivery (Table 2 and 3).

**Table 2:** Background characteristics among women with and without retained placenta

Maternal Characteristics	Non-retained Placenta (*n* = 31)		Retained Placenta (*n* = 29)		*p*-value^a^
Age, median (IQR)	30	6	31	5	ns
Age ≥35, n (%)	4	12.9%	5	17.2%	ns
Previous parity, n (%)					
None	17	54.8%	23	79.3%	ref
1	11	35.5%	5	17.2%	ns
>/=2	3	9.7%	1	3.4%	ns
Previous miscarriages, n (%)					
None	27	87.1%	21	72.4%	ref
1	2	6.5%	6	20.7%	ns
≥ 2	2	6.4%	2	6.9%	ns
Previous abortions, n (%)					
None	23	74.2%	22	75.9%	ref
1 or 2	5	16.1%	4	13.8%	ns
≥ 2	3	9.7%	3	10.3%	ns
Previous cesarean section, n (%)	1	3.2%	3	10.3%	ns
Previous retained placenta, n (%)	2	6.5%	0	0	nc
Assisted reproductive therapy, n (%)	2	6.5%	1	3.4%	ns

^a^ns is *p*-value > 0.05, *nc* not computable

**Table 3:** Delivery-related characteristics among women with and without retained placenta

Delivery-related characteristics	Non-retained placenta (*n* = 31)		Retained Placenta (*n* = 29)		*p*-value^a^
Gestational age, median (IQR)	40 + 1	15	40 + 5	12	ns
Induction of labor^b^, n (%)	5	16.1%	6	20.7%	ns
Epidural analgesia, n (%)	22	71%	22	75.9%	ns
Labor augmentation duration, median (IQR)	2h7min	3h46min	4h23min	4h13min	0.03
Labor augmentation, n (%)	24	77.4%	22	75.9%	ns
Instrumental vaginal delivery, n (%)	1	3.2%	5	17.2%	ns
Labor duration, median (IQR)	8h30min	6h12min	10h45min	7h11min	ns
Labor duration > 12 h, n (%)	7	23.2%	12	41.4%	ns
Fetal weight in g, median (IQR)	3590	500	3627	770	ns
Placental weight in g, median (IQR)	482	102	445	137	ns
Blood loss in ml, n (%)	400	200	1550	1325	<0.001
Blood loss ≥ 1000 ml, n (%)	1	3.2%	23	79.3%	<0.001

^a^ns is *p*-value > 0.05

^b^all women were induced with misoprostol

Median duration of labor augmentation was longer in deliveries with retained placenta (4 h 23 min vs 2 h 07 min; *p* = 0.03) as was median duration of labor (10 h 45 min vs 8 h 30 min; *p* = 0.13). Median blood loss in cases of retained placenta was 1600 ml compared to 400 ml in the group without retained placenta (*p* < 0.001). There was no significant difference in fetal birthweight or placental weight between deliveries with and without retained placenta (Table 3).

### NFκB and IκBα gene and GPX1 protein and gene expression in retained and non-retained placenta

NFκB and IκBα mRNA and GPX1 protein and mRNA were detected at all placental sampling sites. Periumbilical and peripheral GPX1 median protein concentrations were lower in retained placentas compared to non-retained placentas (13.32 ng/ml vs 17.96 ng/ml in periumbilical samples, *p* = 0.22 and 13.27 ng/ml vs 19.09 ng/ml in peripheral samples, *p* = 0.07) but the difference was not statistically significant or only approached statistical significance. Median periumbilical and peripheral GPX1 gene expression were higher in retained placentas (1.13 vs 0.88 for periumbilical samples and 1.32 vs 1.18 for peripheral samples) which approached statistical significance for periumbilical sample (*p* = 0.08) but was statistically non-significant in peripheral samples (*p* = 0.47). There was no significant difference in median periumbilical or peripheral NFκB and IκBα gene expression between retained and non-retained placentas. Median mRNA values for NFκB, IκBα and GPX1 and median protein concentrations for GPX1 with interquartile ranges in retained and non-retained placentas are presented in Table 4.

**Table 4:** Median protein concentration or gene expression of GPX 1, NFκB and IκBα in retained and non-retained placenta

	Non-retained placenta (*n* = 31)		Retained placenta (*n* = 29)		Median difference *p*-value
	Median	Interquartile Range	Median	Interquartile Range	
GPX 1 concentration ng/ml					
Periumbilical	17.96	(7.74–20.07)	13.32	(8.49–17.67)	*p* = 0.22
Peripheral	19.09	(11.81–20.39)	13.27	(3.24–19.18)	*p* = 0.07
GPX 1 mRNA expression^a^					
Periumbilical	0.88	(0.71–1.54)	1.13	(0.91–1.84)	*p* = 0.08
Peripheral	1.18	(0.53–1.65)	1.32	(0.87–1.72)	*p* = 0.47
IκBα mRNA expression^a^					
Periumbilical	0.95	(0.64–1.56)	1.14	(0.74–2.44)	*p* = 0.16
Peripheral	0.99	(0.45–2.64)	1.96	(0.55–5.19)	*p* = 0.25
NFκB mRNA expression^a^					
Periumbilical	0.99	(0.90–1.28)	1.02	(0.82–1.24)	*p* = 0.71
Peripheral	1.04	(0.84–1.22)	1.22	(0.85–1.39)	*p* = 0.17

^a^relative expression to YWHAZ housekeeping gene

### Risk of retained placenta according to level of GPX1 protein concentration

Women with retained placenta were more likely to have a low level of GPX1 protein concentration in placental tissue compared to women without retained placenta (OR 3.82, *p* = 0.02 for periumbilical samples and OR 3.95 *p* = 0.02 for peripheral samples.) This association was strengthened when the analysis was adjusted for duration of labor augmentation (aOR 7.00, *p* = 0.01 for peripheral samples and aOR 3.54, *p* = 0.08 for periumbilical samples).

**Fig. 1:**
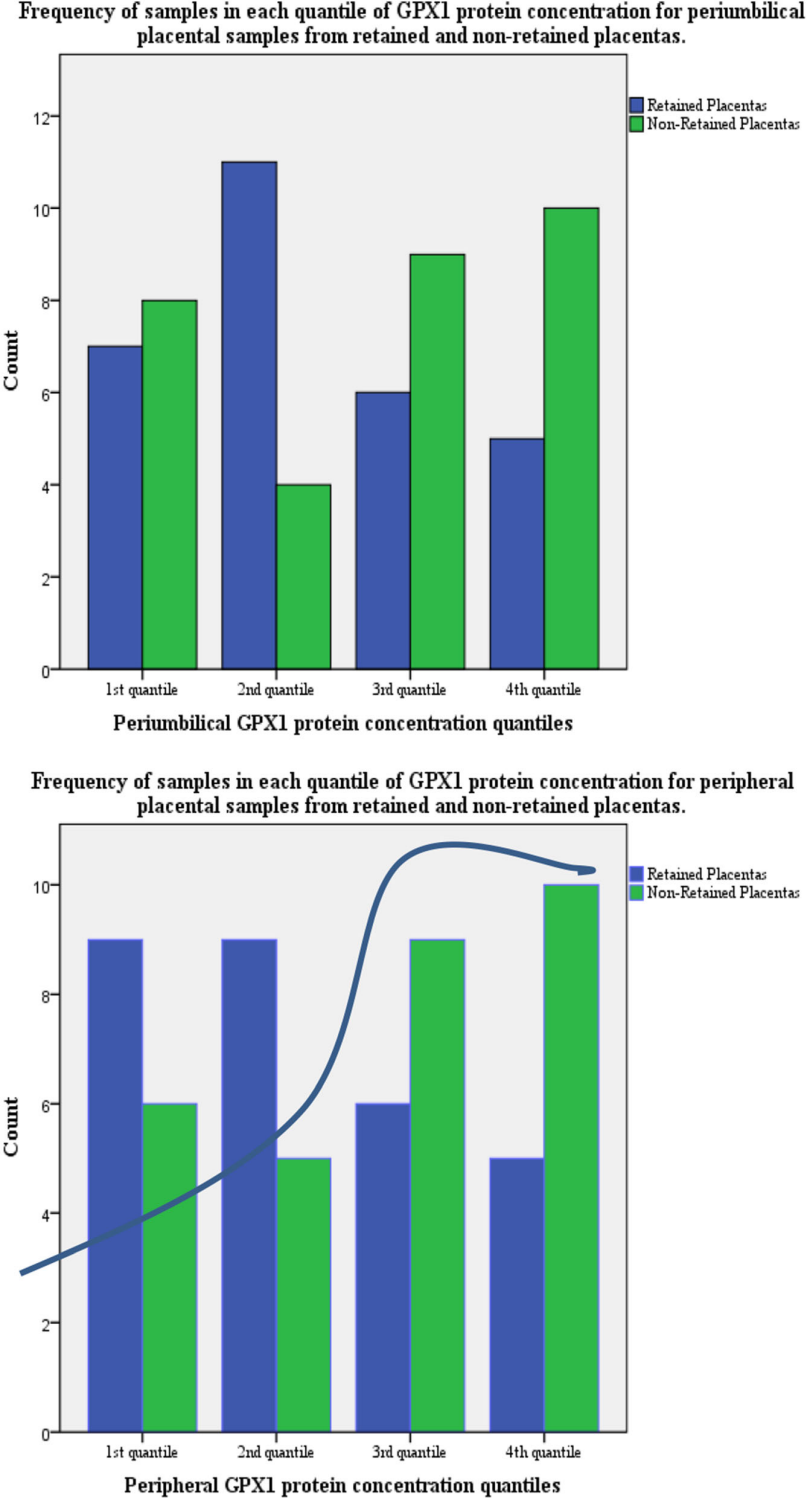
Frequency of samples in each quartile of GPX1 protein concentration for periumbilical and peripheral placental samples from retained and non-retained placentas. * superimposed line showing hypothetical normal GPX1 response to labor

Odds ratios for retained placenta according to GPX1 protein concentration level are presented in Table 5. The frequency of retained and non-retained samples in each quantile of GPX concentration is shown in Fig. 1.

**Table 5:** Level of GPX 1 protein concentration and risk of retained placenta

	Risk of retained placenta			
	Unadjusted Odds Ratio	*p*-value	Adjusted Odds Ratio^b^	*p*-value
Low value^a^ periumbilical GPX 1	3.82	0.02	3.54	0.08
Low value^a^ peripheral GPX 1	3.95	0.02	7.00	0.01

^a^low-value assessment based on sensitivity-specificity analysis of ROC curve. Periumbilical GPX 1 cut-off = 15.62 ng/ml, peripheral GPX 1 = 19.90 ng/ml

^b^adjusted for duration in minutes of augmented labor

### Correlations between GPX1 protein concentration and delivery-related variables

GPX1 periumbilical and peripheral levels did not significantly correlate with duration of labor, duration of labor augmentation or total blood loss. The correlation with duration of oxytocin was the strongest (*r* = 0.33, *p* = 0.12, for peripheral GPX1 levels). Median duration until placental delivery was 1 h 22 min for retained placentas and 08 min 13 s for spontaneously released placentas (*p* < 0.001). Duration until placental release did however not correlate with peripheral or periumbilical GPX1 protein concentrations in either group.

## Discussion

Women with retained placenta were significantly more likely to have a low level of GPX1 protein concentration in placental tissue compared to women without retained placenta. Retained placental tissue showed a tendency of lower median concentrations of GPX1 protein concentration and increased GPX1 gene expression although the differences were not statistically significant. The pattern of decreased expression of GPX1 protein and somewhat increased expression of GPX1 mRNA was however consistent across both peripheral and periumbilical samples of retained placentas.

Although this study did not allow for differences in GPX1 gene or protein levels to be stated with a sufficient degree of confidence, the tendency of reduced antioxidative capacity in retained placentas is interesting in the similarity it bears to preeclampsia. Reduced GPX enzyme activity has been noted in several studies of preeclamptic placentas [13, 15, 22, 23]. One of these showed that GPX mRNA was simultaneously reduced however no distinction was made between the different isoforms of GPX [23]. Other studies where GPX mRNA was analyzed found no significant difference between preeclamptic placentas and controls [14, 24, 25]. Physiologically, GPX1 gene expression in human tissue is thought to be upregulated in response to oxidative stress, in part through an NFκB mediated pathway [12].

It has been suggested that the pattern of increased GPX activity in preeclamptic placentas, in the absence of a concurrent downregulated gene expression, occurs as a result of posttranslational regulation [25]. GPX1 is a selenium dependent protein but selenium deficiency would be expected to affect GPX also at the transcriptional level [26] There are however a number of protein to protein interactions that could modulate GPX protein expression at the posttranslational level [12] which could explain the pattern of normal or possibly increased gene expression and decreased protein expression in retained placentas that our results tentatively suggest.

We found that low GXP 1 concentrations were significantly more common in retained placental tissue. The distribution of biochemical data is often skewed to the left since there are more people with a low to normal value as opposed to a high value which indicates illness. It may be that GPX enzyme concentrations in normal human placenta show the opposite pattern with high to normal values being more common as a result of an adequate physiological response to oxidative stress from pregnancy and labor. It has been suggested that preeclamptic placentas demonstrate lower GPX activity because of an impaired capacity to respond to oxidative stress [14]. Likewise what we may be seeing in the group of retained placentas is the absence of this response.

We adjusted for labor augmentation in a second regression analysis since labor augmentation is associated with retained placenta [20] and has been shown to decrease the GPX substrate glutathione (GSH) indicating increased oxidative stress [27]. Duration of labor augmentation could also act as a proxy variable for labor duration which has also been shown to induce oxidative stress in the placenta [28], although another study has suggested that increased oxidative stress and upregulation of antioxidative defence may be mainly localized to the fetal membranes [14].

It has been shown that a gradient of decreasing oxygenation exists across the placenta from periumbilical to peripheral tissue [29] with the periphery more prone to histological signs of underperfusion [30]. Although we noted a trend of higher gene and protein expression in peripheral samples of all markers studied, the difference in median expression was not significant.

We found no significant differences between gene expression of NFκβ and Iκβ alpha between retained and non-retained placentas. The understanding of NFκβ as a regulator of oxidative stress and inflammation is relatively new [18]. Gene expression of NFκβ and Iκβ alpha has not been studied in preeclamptic placenta to our knowledge. It has however been shown that pro-inflammatory processes in the placenta that occur in response to oxidative stress in vitro are in large part mediated by NFκβ pathways and it has therefore been suggested to play a role in the pathophysiology of this disorder [16]. This study did not suggest an upregulation of NFκβ in retained placenta but the results alone are insufficient to preclude a role for inflammation in the development of this disorder. A closer analysis of inflammation in retained placenta should also include “downstream” products of activated inflammatory pathways such as tissue tumor necrosis factor-alpha, placental interleukins, cyclooxygenase-2, and markers of apoptosis as well as other triggers of inflammatory response and endothelial dysfunction such as soluble fms-like tyrosine kinase-1 (sFlt-1) [16, 31].

The main strength in the study is the homogeneity of the study group. All samples, both retained and non-retained, came from healthy full term pregnancies after vaginal delivery which decreases the confounding effect that differing incidence of other placental disorders, gestational age and mode of delivery between groups would have had on the outcomes studied. Samples were also frozen within a short interval minimizing the effect of exposure to air on oxidative stress levels in the placenta.

There was a large inter-sample variability in GPX1 protein concentrations and relative mRNA expression in our study material which limited the precision of our results. By definition our placentas were collected after labor, a process that may influence levels of oxidative stress per se, as discussed above, [14] but may arguably also increase variability in measurements. Few, if any, other studies on the biochemistry of retained placenta exist. This pilot study is the first to test the hypothesis of increased oxidative stress and inflammation in retained placenta and is consequently limited in the scope of its analysis. Only GPX1 out of at least 4 GPX subtypes relevant to the study of the placenta was analyzed. A more substantial assessment of oxidative stress in relation to retained placenta would require analysis not only these other subtypes but also other antioxidative enzymes as well as by-products of oxidative stress in placental tissue. GPX1 is however the main enzyme isoform in the placenta and is also a vital antioxidative enzyme, as illustrated by the fatal effect of severe oxidative stress on GPX1 knock-out mice [32]. We assessed protein concentration of GPX1 but not enzyme activity. Given that we only analysed GPX1 protein concentration, we chose not to assess enzyme activity as this measurement does not distinguish between isoforms of GPX which would make interpretation and correlation analysis difficult. A further limitation is inherent to the diagnosis itself and will be a constraint in all studies of retained placenta. Retained placentas have by definition been adherent to the uterine wall at least 30 min before placental delivery and almost all cases receive full anesthesia during manual removal of the placenta. We can only speculate as to the effect that these factors have on the levels of oxidative stress and inflammation in the placenta. Our sensitivity analysis however showed no correlation between duration till placental release and GPX1 protein levels.

If retained placenta has both epidemiological and biochemical properties in common with preeclampsia this might suggest that retained placenta is not a random occurrence but part of a spectrum of placental disorders and that it is to some extent predictable. Further studies of the role of oxidative stress in retained placenta might be instrumental in the prevention or less invasive treatment of this disorder.

## Conclusions

Women with retained placenta were more likely to have a low level of GPX1 protein concentration in placental tissue compared to women without retained placenta and retained placental tissue showed a tendency of lower median concentrations of GPX1 protein expression. The results are statistically uncertain but, given the similarity to patterns of decreased GPX activity seen in several studies of preeclamptic placentas, may warrant further investigation into the relation between retained placenta and this disorder and a discussion of the role of oxidative stress in the pathophysiology of retained placenta.

## Abbreviations

aOR: adjusted odds ratio
ELISA: Enzyme Linked-Immunosorbent Assay
GPX1: Glutathione Peroxidase 1
IQR: Interquartile range
Iκβα: Nuclear Factor kappa light polypeptide gene enhancer in B-cells inhibitor, alpha
NFκB: Nuclear Factor κappa βeta
OR: Odds ratio
qRT-PCR: Real-time quantitative reverse transcriptase polymerase chain reaction analysis
RNA: Ribonucleic acid
ROC: Receiver Operating Characteristic
